# Classes in Translating and Interpreting Produce Differential Gains in Switching and Updating

**DOI:** 10.3389/fpsyg.2016.01297

**Published:** 2016-08-30

**Authors:** Yanping Dong, Yuhua Liu

**Affiliations:** ^1^Bilingual Cognition and Development Lab/Center of Linguistics and Applied Linguistics, Guangdong University of Foreign StudiesGuangzhou, China; ^2^College of Foreign Studies, South China Agricultural UniversityGuangzhou, China

**Keywords:** cognitive control, processing demand, translation, interpreting, bilingual advantage, longitudinal study

## Abstract

The present longitudinal study was intended to investigate whether the two bilingual experiences of written translation and consecutive interpreting (featured with similar language switching experience but different processing demands) would produce different cognitive control effects in young adults. Three groups of Chinese–English young adult bilinguals, who differed mainly in their half-year long bilingual experience: one for general L2 training, one for written translation and one for oral consecutive interpreting, were tested twice on the number Stroop, switching color-shape and N-back tasks. The results show that the interpreting experience produced significant cognitive advantages in switching (switch cost) and updating, while the translating experience produced marginally significant improvements in updating. The findings indicate that the experience of language switching under higher processing demands brings more domain-general advantages, suggesting that processing demand may be a decisive factor for the presence or absence of the hot-debated bilingual advantages.

## Introduction

It is believed that pervasive experience can leave its mark on the development of mind and brain. The past decade has seen a boom of research exploring the effect of bilingualism on specific cognitive control components (e.g., Bialystok et al., 2004). But there have been dissenting voices (e.g., Paap and Greenberg, 2013) or cautious voices (e.g., Hilchey and Klein, 2011) in recent years. This controversy has turned into a hot debate, especially after a recent issue of Bilingualism: Language and Cognition (a series of commentaries on the key article by Valian, 2015) and a recent issue of Cortex (a series of commentaries on the key article by Paap et al., 2015). Experts on the topic have expressed their warnings against methodological flaws (see Paap, 2014, for example), theoretical weaknesses (see Jared, 2015, for example), and interpretation biases (see Morton, 2015, for example). Aware of the controversy, the present study has taken several steps to overcome some of the flaws and weaknesses in the literature, hoping to find a way forward, which may provide some clues for the bilingual advantage issue, and which may further help to establish the types of bilingual experience that produce relatively quick gains in cognitive control.

A large majority of research on the bilingual advantage adopted a cross-sectional design (except for a few such as Bak et al., 2014). However, cross-sectional, in contrast to longitudinal designs, are vulnerable to confounding factors that are hard to control and for which the cause-consequence relationship between bilingualism and executive control is hard to decide (see Kempe et al., 2015; Li and Grant, 2015; Woumans and Duyck, 2015).

Indeed, many bilingual advantages have been reported using assumed measures of inhibition, switching and monitoring, but it seems that many of them have been questioned by Paap et al. (2015) for reasons illustrated above. The inhibition advantage from bilingualism was evidenced in different tasks, such as the Simon task (e.g., Bialystok et al., 2004; Carlson and Meltzoff, 2008; Woumans et al., 2015), the Stroop task (e.g., Bialystok et al., 2008; Blumenfeld and Marian, 2011), the flanker task (e.g., de Abreu et al., 2012; Poarch and Bialystok, 2015) and the Attention Network Test (ANT; a complex version of flanker) (e.g., Costa et al., 2008; Marzecová et al., 2013). Bilingual advantage in switching was shown in the color-shape task (e.g., Prior and Macwhinney, 2010; Prior and Gollan, 2011). As to the relationship between bilingual experiences and updating capacity, few empirical studies have been conducted, but there have been theoretical formulations on the relationship between WM and updating in the context of bilingual advantage (Paap and Sawi, 2014). A few studies found that bilingualism did not bring WM advantage as measured by WM spans (e.g., Ratiu and Azuma, 2015). Bilingual advantage in monitoring (as indicated by shortened reaction times in tasks containing conflicts or by mixing cost in the color-shape task) was reported too (e.g., Barac and Bialystok, 2012; Abutalebi et al., 2015; Woumans et al., 2015). However, null bilingual effects have also been reported in the Simon task (e.g., Gathercole et al., 2014; Kirk et al., 2014), the Stroop task (e.g., Kousaie and Phillips, 2012) and the flanker task (e.g., Bialystok et al., 2010). The bilingual switching advantage failed to appear in some studies either (e.g., Hernandez et al., 2013; Gathercole et al., 2014). Reviewing nearly 30 experiments, Hilchey and Klein (2011) claimed that there was only evidence for a bilingual advantage in monitoring. However, Paap and Greenberg (2013), after reviewing 18 tests in several studies, did not find any significant monitoring advantage. The bilingual advantage issue, therefore, needs more research, especially research adopting a longitudinal design.

Apart from methodological considerations, a better theoretical framework is needed (e.g., Jared, 2015; Hartsuiker, 2015). The most important question is: what does bilingualism have that monolingualism does not that might lead to bilingual advantages in cognitive control? The general theoretical formulation is that executive functions exercised in selecting the target language during bilingual processing (see the BIA+ model by Dijkstra and Van Heuven, 2002; the Inhibitory Control Model by Green, 1998) are transferred from the linguistic domain to the general domain. It seems that the monitoring of two jointly activated language systems, the inhibition of the non-target language, the switch between languages, and the updating of relevant information in the bilingual language control system corresponds neatly to such components of the general cognitive control system as monitoring, inhibiting, switching and updating (see Miyake et al., 2000). But how does this transfer happen? In Hartsuiker’s (2015) words, when, how and why does practice in one domain generalize to another domain? Hartsuiker (2015) may have pointed out the most important direction for future research, and the present paper was intended as a first step in the recommended direction.

Instead of investigating the bilingual advantage directly, the present study investigates a related issue: under what circumstances does language switching practice start to influence or enhance non-linguistic switching abilities? The answer to this question could partly answer Hartsuiker’s (2015) question of when practice in one domain generalizes to another domain. Interpreting between two languages is a cognitively demanding task, and several recent studies (Yudes et al., 2011; Dong and Xie, 2014; Babcock and Vallesi, 2015; Morales et al., 2015; Woumans et al., 2015; Becker et al., 2016) have explored how interpreting experience brings cognitive advantages. Yudes et al. (2011) found that professional simultaneous interpreters (SIs) outperformed general bilinguals in the WCST task, but not in the task of Simon. Consistent with these findings, Dong and Xie (2014) further found that students of interpreting training or more interpreting training outperformed those of no or less interpreting training in the task of WCST, but not in the task of Flanker. Babcock and Vallesi (2015) and Woumans et al. (2015), however, had different findings. Babcock and Vallesi (2015) found that professional interpreters exhibited less mixing cost in a color-shape task than general bilinguals but did not show advantages in conflict resolution in a Stroop task or switching cost in the color-shape task. Woumans et al. (2015) found that interpreters outperformed unbalanced (but not balanced) bilinguals in the Simon and ANT tasks (i.e., higher accuracy in both tasks and smaller error congruency effect in the ANT). Along the same line of comparing SIs and general bilinguals, Morales et al. (2015) reported higher updating skills from SIs and a modulating effect of interpreting experience on the interaction between attentional networks. Comparing SIs and other professional multilingual controls (mostly consecutive interpreters and translators), Becker et al. (2016) reported less mixing costs in a color-shape switching task and a dual-task advantage from SIs. To sum up, in the few cross-sectional studies conducted up till now, it seems that there was always a certain cognitive control advantage for professional SIs or students of more interpreting experience. However, the results were not necessarily consistent. Two of the studies (Yudes et al., 2011; Dong and Xie, 2014) found that interpreting experience enhanced switching ability as measured in the WCST, while two of the studies (Babcock and Vallesi, 2015; Becker et al., 2016) found that interpreting experience reduced mixing costs but not switching costs in a color-shape task. To bridge the gap, we may have to conduct studies of a longitudinal design and with both tasks (WCST and the color-shape task). What is more, we have to take into consideration of our critical question of when (under what circumstances).

To answer the critical question of when language switching practice starts to influence or enhance non-linguistic switching abilities, the present paper adopts a longitudinal design and compares the cognitive consequences of (oral consecutive) interpreting training with (written) translation training and general second language training. Three groups of bilingual students participated and they were comparable except that they would respectively receive one semester’s consecutive interpreting training, translation training and general L2 training (L2 culture and communication). Apart from the longitudinal design, what is distinctively different from the literature is a comparison with translation training. On the one hand, performances of both interpreting and translation involve frequent switching between two languages. Different from simultaneous interpreting, consecutive interpreting is more “serial” in the sense that it is generally after one segment of the source text is rendered that the next would start to be processed. It is in this sense that consecutive interpreting is more similar to translation, compared to simultaneous interpreting. On the other hand, there are differences between consecutive interpreting and translation. The most apparent difference lies in that interpreting requires immediate processing, which suggests that interpreters are under great time pressure and that they have to store on-line a huge amount of information. Dragsted and Hansen (2009) found that because of this difference, professional translators and interpreters performed differently in an eye-tracking experiment of sight translation and written translation. The interpreters translated faster in a more “controlled” linear way without compromising output quality, while the translators translated more slowly with plenty of backtracking and regressions of their eye movements. Yudes et al. (2011) and Dong and Xie (2014) have found evidence for the cognitive advantage of switching brought by interpreting experience, but none of them explicitly distinguished oral interpreting experience from written translation experience because students of interpreting (as in Dong and Xie, 2014) or professional interpreters (as in Yudes et al., 2011) are generally also trained in written translation. A direct comparison of the cognitive effects of these two modes of language training may be able to provide some clues for why some language experiences rather than others bring cognitive control advantages, and thus clues for what brings bilingual cognitive advantages.

We predicted that interpreting experience would bring more cognitive control advantages than translation or general bilingual experience. If the prediction is true, it implies that a prerequisite for a certain training to bring about general cognitive control advantage is high processing demands. For the interpreting-translation case, immediate switching of a large chunk of speech (a sentence at least) between the two languages under time pressure (i.e., interpreting) poses higher processing demands than switching without time pressure (i.e., translation). A task may be demanding in different ways, but immediate processing under time pressure is certainly one of the ways. As speculated by Schroeder and Marian (2016), when the supply was below the demand, the cognitive system tried to adapt and thus got enhanced. Therefore, the answer for the critical question of when would be: language switching practice starts to influence or enhance non-linguistic switching abilities when processing demands reach a certain level.

## Materials and Methods

To investigate how the two specific bilingual experiences of translation and interpreting would influence cognitive control development in young adults, three groups of Chinese–English bilingual participants were tested at a pre-test and a post-test. The three groups were comparable except that one group would receive one semester’s (oral) interpreting training, another (written) translation training and the third would receive general L2 training (English culture and communication). There were two parts in the pre-test: (1) a questionnaire of the participants’ backgrounds: their L2-related experiences and their relevant biological and social data (e.g., age, IQ, parents’ education); (2) a test of their cognitive control abilities of inhibition, switching, monitoring and updating in working memory (WM). The post-test consisted of only the second part, that is, a test of participants’ cognitive control abilities. Statistical analyses reported below will show how each group has progressed after one semester’s training, and how the three groups differ from each other in cognitive control abilities after being matched in their pre-test.

### Participants

Three groups of Chinese–English young adult unbalanced bilinguals (145 in total, mean age = 19.69 years, *SD* = 0.89, range = 17–22) volunteered to participate in the study for course credit. Among the 145 participants, 57 of them taking an interpreting course during the experiment semester (coded henceforward as the interpreting group), 43 of them taking a translation course (coded henceforward as the translation group), and 45 of them taking general English course (English culture and communication, coded henceforward as the control group). All these participants were non-English-major sophomore students from the same college of a Chinese university in China, and received neither translation nor interpreting training before taking the pre-test. Since the courses were elective, assignment to the groups was based on self-selection. In the general English course (control group), about half of the class time was spent on listening to the teacher’ lectures and half on student discussions. Teachers and students were all required to speak in English in the classroom and therefore little language switching took place. As for the two courses of translation and interpreting, the training was mainly from English to Chinese, with about one third of the class time spent on listening to teachers’ lectures and the rest on translation or interpreting practice. At the end of the semester, participants were asked to report how much time they had spent on each course after class. The average time each group of participants spent on Integrated English after class was 56 h, and that on their distinguishing course (English culture and communication, translation or interpreting) was 40 h.

A comparison of the courses that the participants received during the experimental semester is illustrated in **Table 1**. The three groups were, therefore, comparable in the training they received during the semester except for the difference deliberately designed for the present study.

**Table 1:** Class hours of courses for the three participant groups (control, translation and interpreting) together with practice after class (in brackets) during the experimental semester.

	Control	Translation	Interpreting
Courses not related to L2 (English)	256	256	256
Integrated English	42 (+56)	42 (+56)	42 (+56)
English culture and communication	32 (+40)	0	0
Translation (written)	0	32 (+40)	0
Interpreting (oral)	0	0	32 (+40)

All the participants were native speakers of Chinese, and apart from English, had no contact with any other foreign language. Details of their background information were presented in the first half of **Table 2** “background characteristics,” including L2-related factors (tested L2 proficiency, self-rated L2 proficiency, self-rated L2 use, AoA) and more biological and social factors (age, IQ, parents’ education). Such information was collected to ensure that confounding factors (e.g., Dong and Li, 2015; Valian, 2015) would be controlled.

**Table 2:** Pre-test group means (with SD) and comparisons (*p*-value) of participants’ background characteristics and task performances in the pre-test before group match.

	Control (*n* = 43)	Translation (*n* = 40)	Interpreting (*n* = 51)	*p*-Value
Background characteristics				
Translation/interpreting	No	No	No	
Tested L2 proficiency	14.13 (3.61)	13.93 (4.47)	13.39 (3.37)	0.614
Self-rated L2 proficiency	20.02 (4.37)	21.07 (5.74)	20.76 (5.27)	0.630
Self-rated L2 use	0.054 (0.036)	0.048 (0.047)	0.049 (0.044)	0.731
AoA	8.95 (2.31)	9.17 (2.44)	9.00 (2.32)	0.903
Age	19.81 (0.82)	19.80 (0.99)	19.45 (0.83)	0.079
Father education	2.39 (0.69)	3.15 (1.38)	2.80 (1.24)	0.013
Mother education	2.02 (0.98)	2.72 (1.37)	2.33 (1.21)	0.031
Intelligence	67.62 (2.38)	67.05 (2.71)	66.66 (3.11)	0.250
Cognitive control abilities				
Stroop: global RTs (ms)	684.71 (84.73)	677.66 (61.75)	670.69 (68.79)	0.646
Stroop: Stroop effect	34.72 (36.10)	31.36 (49.39)	17.79 (37.08)	0.105
Stroop: Stroop inhibition	14.51 (44.48)	6.27 (56.64)	-2.73 (36.43)	0.192
Stroop: Stroop facilitation	-20.21 (36.80)	-25.09 (31.86)	-20.52 (42.65)	0.804
Color-shape: global RTs	612.57 (141.95)	598.62 (145.89)	587.51 (124.12)	0.676
Color-shape: mixing cost	130.44 (126.70)	103.18 (111.06)	117.39 (90.24)	0.526
Color-shape: switch cost	148.30 (95.22)	123.24 (83.72)	137.77 (85.82)	0.435
N-back: global RTs	840.51 (265.32)	848.20 (248.04)	857.64 (248.27)	0.948
N-back: accuracy rate	0.86 (0.088)	0.87 (0.083)	0.83 (0.097)	0.108

### Materials and Tasks

Critical information about the materials and tasks is listed in **Table 3**.

**Table 3:** Summary of tasks used in the present study and description of the items tested.

Tasks	Items tested
*Tasks capturing background (in pre-test only)*	
Composite questionnaire	(1) Self-rated language proficiency: overall score of listening, speaking, reading and writing respectively on a 10-point Likert scale; 40 points in total (2) Self-rated language use: percentage of daily language use; (3) AoA: age of English education; (4) Age: age when being tested; (5) Parental education: score of parents’ education on a 5-point Likert scale
L2 cloze test	L2 proficiency (Bachman, 1985), 30 points in total
IQ test	IQ: Raven’s Advanced Progressive Matrices Set (Raven et al., 1977), 72 points in total
*Cognitive control tasks (in pre- and post-tests)*	
Number Stroop task	(1) Inhibition ability: Stroop conflict (2) Monitoring ability: global RTs
Color-shape task	(1) Switching ability: switch cost (2) Monitoring ability: mixing cost and global RTs
N-back task	(1) Updating ability: accuracy rate, global RTs

In the pre-test, participants had to complete a composite questionnaire with questions tapping information about participants’ self-rated L2 proficiency, self-rated L2 use, AoA, age and parental education (Marian et al., 2007), together with an L2 proficiency test (L2 cloze test by Bachman, 1985) and an IQ test (Raven et al., 1977).

Altogether three tasks of cognitive abilities were used, testing participants’ inhibition, switching, updating, and monitoring. Inhibitory control was tested with the number Stroop task under the typical assumption that smaller Stroop interference effects reflect better control. We did not choose the Simon task or the Flanker or the color Stroop because we believed they were too simple for our young adult participants who were in their peak of cognitive abilities (e.g., Paap and Greenberg, 2013). Xie and Dong (2015) used the Flanker and the number Stroop tasks to test similar participants (Chinese–English young adult unbalanced bilinguals with L1 or L2 public speaking training) and it was found that the number Stroop task produced more groups effects than the Flanker, probably because it was more difficult (with longer reaction times, see Dong and Li, 2015 for a review). But we are aware that there may be different opinions. Paap et al. (2014, May) reported that the flanker effect is still shrinking after 100 sessions and more than 20,000 trials.

Switching (mental set shifting or mental flexibility) was tested with the color-shape task (i.e., the switch cost: reaction time difference between a switch trial and a non-switch trial in a mixed block)^1^. Both global RTs and mixing costs (RT difference between non-switch trials in a mixed block and single task trials) are often assumed to reflect monitoring ability. But we are aware that “switch cost” is also taken as a measure of inhibitory control, in the sense that participants have to inhibit the previous task set to be able to reactivate the new one (Philipp et al., 2008; Yang et al., 2016). And yet based on the tripartite system of executive functions suggested by Miyake et al. (2000), we decided to adopt the switching account of switch cost as adopted in Hernandez et al. (2013) and Paap and Greenberg (2013). The major reason is that compared with the inhibition component measured in tasks such as the Flanker, the Simon and the Stroop, switch cost in the color-shape task involves more of one’s ability to switch to a new task set.

In addition to the two typical components of inhibition, switching and monitoring, updating in WM was also identified as part of the cognitive control system (e.g., Miyake et al., 2000; Costa et al., 2009; Hilchey and Klein, 2011). These components are related but also relatively independent. The enhancement of one component may or may not imply the strengthening of other components. Thus, all four components were tested in the present study (see **Table 3**).

Each cognitive control task is described in more detail below.

#### The Number Stroop Task

The number Stroop task, measuring participants’ inhibition ability, was more or less the same as that used by Xie and Dong (2015). The task required participants to judge whether the number of the digits or the hash signs (#, ##, ###, or ####) in a stimulus was even or odd. There were three possible conditions. The neutral condition refers to trials of the hash sign “#”, and so the correct response for “###” or “#”, for example, would be odd. The congruent condition refers to trials of digits in which the parity of the digit coincides with the parity of the number of the digit, and so the correct response for 2222 would be even. The incongruent condition is the opposite of the congruent condition, and so the correct response for 222 would be odd (because there are three digits). The computerized task was composed of two blocks: the practice block and the experimental block. The practice block consisted of nine trials with feedback of accuracy and response times for each stimulus. The experimental block consisted of 120 randomly presented trials, with 40 in each condition. Each stimulus was presented on the screen for a maximum time of 2000 ms or until participants pressed designated keys. Participants were asked to respond as quickly as possible without sacrificing accuracy.

We computed four indices for the Stroop task: Global RTs, Stroop effect, Stroop facilitation and inhibition (see **Table 2**). The most important one is the Stroop effect, i.e., the difference in mean RTs between incongruent trials requiring suppression of conflicting cues and congruent trials with no conflicting cues. A smaller Stroop effect implies higher ability in conflict resolution and inhibition. Global RTs refers to the average time taken to respond to all the trials (congruent, neutral and incongruent trials). Stroop facilitation refers to the RT difference between congruent and neutral trials, while Stroop inhibition refers to the RT difference between incongruent and neutral trials.

#### The Color-Shape Switching Task

The color-shape task was adapted in the present study so that the inhibition component in switch costs was reduced. In a typical manipulation of the color-shape task, the stimulus is one of the four combinations of color and shape: red/green circle/square. A precue is therefore necessary to indicate when to respond to color and when to respond to shape. But as in the number Stroop task, a single shape contains both cues of color and shape. To respond to color, for example, one has to inhibit a potential response to shape. The present study instead tried to reduce the component of inhibition in the color-shape task in which the stimulus was either one of the two color pictures (red or green) or one of the two colorless shapes (circle or triangle).

Designed deliberately to test participants’ switching ability, the color-shape task required participants to press the designated keys corresponding to color (always in a circle) or colorless shape pictures presented at the center of the computer screen. Each trial started with a fixation cross presented at the center for 350 ms, followed by a blank screen for 150 ms, and then the target appeared and remained at the center until the participant responded. There were four choices of target picture: two color pictures (red circle or green circle) and two shape pictures (circle or triangle without any color). Participants were instructed to perform the color task using the left hand, with “red” being assigned to the index finger, and “green” the middle finger. The shape task was performed with the right hand, with “triangle” being assigned to the index finger and “circle” the middle finger. The experiment was composed of three blocks: two blocks of a single task (color or shape) and one block of the mixed task (color and shape). Each single task block included 8 practice trials followed by 24 experimental trials, and the mixed task block included 8 practice trials followed by 48 experimental trials. All the trials in each block were randomized. Participants were asked to respond as quickly as possible without sacrificing accuracy.

Three indices were computed for the color-shape task: global RTs, mixing cost and switch cost. Global RTs refers to the mean RTs in the mixed task block. Mixing cost refers to the difference in RTs between the non-switch trials in the mixed task block and trials in the single task block, while switch cost refers to the difference in RTs between the switch trials and non-switch trials in the mixed task block. Both global RTs and mixing cost are indicators of monitoring ability, and switch cost indicates the ability to switch between different types of trials.

#### The N-back Task

A visuo-spatial version of the 2-back task was used to measure updating in WM. In the 2-back task, a blue square was presented in one of 25 possible locations on the screen. Participants were asked to match the location of the current square with the location of the square before the previous one (2-back). The task consisted of 42 2-back trials (28 non-target and 14 target trials). Participants were asked to press the “F” button if the square was in the same location as the square two trials back and the “J” button if the location was different. The square remained on the screen for 500 ms. A new square appeared 3000 ms after the previous one had disappeared, irrespective of whether a response was made or not. The presentation order of the trials was randomized. Before the experimental sequence, participants were asked to complete three practice sequences of 27 trials. Participants were asked to respond as quickly as possible without sacrificing accuracy. Two indices, i.e., global RTs and accuracy rate, were computed and both were indicators of participants’ updating ability.

#### Procedure

The experiment lasted for one academic semester (about 4 months and a half). At the beginning of the semester, participants were asked to take the pre-test in a computer room. The test was divided into two parts and lasted for nearly 2 h, with a 5-min break in between. The order of task administration was fixed for all three groups, with the requirement that no two tasks tapping the same cognitive control capacity occurred consecutively so as to minimize any error caused by task interference. Based on this criterion, tasks administered in the first part were the questionnaire, the number Stroop task and the color-shape switch task. Those in the second part were the cloze test, the N-back task and the IQ test.

As illustrated in the section of “participants,” after the pre-test, participants as college students took various courses for one semester. At the end of the semester, participants took the post-test. Similar to the pre-test, the post-test was divided into two parts and the tasks were administered in a fixed order for all the groups, those in the first part were the number Stroop and the color-shape tasks, and those in the second part were the cloze test and the N-back task. Before the first part started, participants were asked to complete a questionnaire to collect information about their experiences in the past semester.

## Results

### Data Trimming

First of all, we had to exclude those participants whose performances were obviously not normal. The reason is that some of the students were not serious enough, at least in some of the tests. The three courses were selective, and students are generally not as serious in a selective course as when they perform tasks in a compulsory course. What is more, the classes were very large (i.e., respectively 45, 43, and 57 students in each class), especially as language classes, and it is generally harder to ensure that all students are serious enough in a large class. **Table 4** lists the number of participants excluded from each group of participants, and the reasons for the exclusions.

**Table 4:** Number of participants excluded from further data analysis and reasons for the exclusions.

	Control 45-2	Translation 43-3	Interpreting 57-6
Computer breakdown in n-back post-test		1	
Abnormal performance in L2 test (less than 10 out of a total of 30 and worse in post-test than in pre-test)	1	1	3
Background different from others (nervous as the only first-year student among all second-year students)		1	
Not serious in n-back post-test (wrong input of her student number, lowest accuracy at 69%)	1		
Abnormal performance in IQ test (less than 55 out of a total of 72, which means “retarded” according to Raven et al., 1977).			3

The data were trimmed following general procedures in the literature. In the number Stroop task, data from erroneous responses and data with response time (RTs) less than 200 ms were first discarded, and then outlier responses deviating by more than 3 SDs from the mean RTs for each participant were trimmed. Altogether less than 5% of data was discarded (the control group: pre-test, 1.91%; post-test, 2.12%; the translation group: pre-test, 2.21%; post-test, 2.14%; the interpreting group: pre-test, 1.97%; post-test, 1.86%).

In the color-shape task, the same procedure was followed, and less than 5% of data was discarded (the control group: pre-test, 1.09 and 1.35% for the single or mixed task block; post-test, 1.06 and 1.67% respectively the two task blocks; the translation group: the four percentages were respectively 1.02, 0.91, 1.07, 1.71%; the interpreting group: respectively 1.02, 1.81, 0.68, 1.41%).

In the N-back task, the same procedure was followed and less than 5% of data was discarded (the control group: pre-test, 1.55%; post-test, 1.38%; the translation group: pre-test, 1.18%; post-test, 1.18%; the interpreting group: pre-test, 1.09%; post-test, 2.24%).

### Statistical Analysis

An analysis was conducted with Participant Group as the between-subject factor and Testing Time as the within-subject factor, hoping to find out whether there were any group differences in the training effect. A prerequisite for the analyses was that the three groups were matched in all the relevant factors that may influence the performance or development in the cognitive control tasks. Details of data analyses are reported below.

#### Raw Data in the Pre-test

Between-group comparisons were conducted for the pre-test results to see whether the three groups were matched or not. **Table 2** is a summary of the descriptive data together with the *p*-value for each group comparison in each index.

The first finding revealed in **Table 2** is that there was no group difference in any of the indices of cognitive abilities, and that there was no group difference in any of the L2-related indices (i.e., L2 proficiency, L2 use and AoA). Since the three courses (oral interpreting, written translation, and general L2 class) were not compulsory and students made their choice out of their own will, *this finding indicates that students did not choose a certain training (e.g., interpreting) because of some preexisting advantage in a related cognitive function (e.g., switching).*

#### Group Matching in the Pre-test

To make sure that group differences in cognitive control abilities in the post-test were indeed caused by the different types of training that the participants had received, not by any preexisting group differences, we conducted a series of regression analyses to see which background characteristics played a significant role in cognitive control abilities in the post-test. Several factors were moderately correlated (father education and mother education: *r* = 0.498, *p <* 0.001; AoA and age: *r* = 0.364, *p <* 0.001; AoA and mother education: *r* = -0.334, *p <* 0.001; self-rated L2 proficiency and tested L2 proficiency: *r* = 0.363, *p <* 0.001), we therefore adopted stepwise regressions to overcome the difficulty in assessing the unique contribution of a variable. The result was that father education significantly contributed to Stroop effect (father education: β = 0.238, *p* = 0.007) and Stroop inhibition (father education: β = 0.180, *p* = 0.040)^2^. The three groups differed in pre-test father education (*p* = 0.022, η^2^ = 0.057), mother education (*p* = 0.031, η^2^ = 0.052) and age (*p* = 0.079, η^2^ = 0.038). A closer look at parents’ education across the groups shows that the translation group enjoyed higher parents’ education than the other two groups. Besides, the interpreting group was the youngest among the three groups. We therefore matched the participant groups on background characteristics and cognitive control abilities in the pre-test mainly in two steps. First, we excluded participants with high parents’ education from the translation group (five participants) and participants with low parents’ education from the control group (five participants) and interpreting group (four participants). Second, we excluded one oldest participant from the control group and three youngest participants from the interpreting group. See Supplementary A for detailed information of the excluded participants. **Table 5** shows the result of the match, with participant groups matched in all the key testing items, especially those of cognitive control abilities (e.g., group match for N-back accuracy rate enhanced).

**Table 5:** Group means (with SD) and comparisons (p value) of participants’ background characteristics and task performances in pre- and post- tests after group match.

	Control (*n* = 37)	Translation (*n* = 35)	Interpreting (*n* = 44)	*p*-Value
Background characteristics				
Translation/interpreting	No	No	No	
Tested L2 proficiency	14.00 (3.70)	14.12 (4.57)	13.36 (3.55)	0.645
Self-rated L2 proficiency	20.43 (4.07)	21.31 (5.69)	20.41 (5.52)	0.694
Self-rated L2 use	0.060 (0.036)	0.050 (0.049)	0.048 (0.045)	0.438
AoA	8.73 (2.41)	9.14 (2.49)	9.20 (2.33)	0.645
Age	19.73 (0.83)	19.85 (1.03)	19.54 (0.73)	0.276
Father education	2.46 (0.66)	2.91 (1.31)	2.82 (1.24)	0.187
Mother education	2.11 (1.02)	2.57 (1.29)	2.34 (1.22)	0.256
Intelligence	67.65 (2.46)	66.86 (2.64)	66.50 (3.22)	0.186
Cognitive control abilities in the pre-test				
Stroop: global RTs (ms)	676.52 (86.17)	682.34 (60.66)	662.88 (65.03)	0.457
Stroop: Stroop effect	34.13 (37.42)	33.59 (51.82)	18.48 (38.35)	0.172
Stroop: Stroop inhibition	14.68 (40.05)	6.40 (59.86)	-1.53 (32.08)	0.267
Stroop: Stroop facilitation	-19.45 (33.75)	-27.19 (32.50)	-20.01 (40.72)	0.598
Color-shape: global RTs	612.30 (148.68)	601.44 (152.75)	581.31 (132.42)	0.616
Color-shape: mixing cost	138.86 (132.39)	103.43 (118.39)	113.48 (93.21)	0.397
Color-shape: switch cost	140.32 (73.49)	122.05 (81.76)	139.23 (90.19)	0.572
N-back: global RTs	855.91 (266.30)	831.97 (225.65)	852.17 (250.49)	0.907
N-back: accuracy rate	0.85 (.091)	0.86 (.080)	0.84 (.091)	0.533
Cognitive control abilities in the post-test				
Stroop: global RTs	629.08 (82.73)	624.97 (46.92)	612.48 (52.45)	0.457
Stroop: Stroop effect	28.03 (28.42)	26.31 (30.71)	31.14 (32.28)	0.776
Stroop: Stroop inhibition	0.29 (40.63)	6.85 (34.46)	9.06 (31.39)	0.526
Stroop: Stroop facilitation	-27.74 (35.09)	-19.46 (30.57)	-22.08 (34.16)	0.542
Color-shape: global RTs	548.03 (83.89)	557.91 (109.72)	529.95 (91.09)	0.414
Color-shape: mixing cost	99.57 (58.73)	110.23 (87.84)	107.75 (74.01)	0.813
Color-shape: switch cost	132.73 (82.93)	118.71 (70.88)	94.20 (58.19)	0.049
N-back: global RTs	876.62 (262.99)	762.40 (192.39)	752.93 (215.11)	0.032
N-back: accuracy rate	0.89 (0.084)	0.91 (0.067)	0.90 (0.068)	0.627

#### Pre-test– Post-test Comparisons across Groups

It is important to know how each group progressed from the pre-test to the post-test and whether groups differed from each other in the degree of progress. Participant Group (between-subject factor) × Test Time (within-subject factor) ANOVAs were therefore conducted. **Table 6** shows the result of analyses.

**Table 6:** Summary of Group × Test Time analyses in each task index of the cognitive control tasks.

	Main effect of Test Time	Main effect of Group	Interaction effect
Stroop: global RTs	*p* < 0.001, $\eta_{p}^{2}$ = 0.475	*p* = 0.416, $\eta_{p}^{2}$ = 0.015	*p* = 0.734, $\eta_{p}^{2}$ = 0.005
Stroop: Stroop effect	*p* = 0.961, $\eta_{p}^{2}$ < 0.001	*p* = 0.519, $\eta_{p}^{2}$ = 0.012	*p* = 0.157, $\eta_{p}^{2}$ = 0.032
Stroop: Stroop inhibition	*p* = 0.839, $\eta_{p}^{2}$ < 0.001	*p* = 0.815, $\eta_{p}^{2}$ = 0.004	*p* = 0.165, $\eta_{p}^{2}$ = 0.031
Stroop: Stroop facilitation	*p* = 0.851, $\eta_{p}^{2}$ < 0.001	*p* = 0.414, $\eta_{p}^{2}$ = 0.015	*p* = 0.852, $\eta_{p}^{2}$ = 0.003
Color-shape: global RTs	*p* < 0.001, $\eta_{p}^{2}$ = 0.196	*p* = 0.511, $\eta_{p}^{2}$ = 0.012	*p* = 0.713, $\eta_{p}^{2}$ = 0.006
Color-shape: mixing cost	*p* = 0.233, $\eta_{p}^{2}$ = 0.013	*p* = 0.785, $\eta_{p}^{2}$ = 0.004	*p* = 0.206, $\eta_{p}^{2}$ = 0.028
Color-shape: switch cost	*p* = 0.014, $\eta_{p}^{2}$ = 0.052	*p* = 0.371, $\eta_{p}^{2}$ = 0.017	*p* = 0.039, $\eta_{p}^{2}$ = 0.056
N-back: global RTs	*p* = 0.012, $\eta_{p}^{2}$ = 0.054	*p* = 0.300, $\eta_{p}^{2}$ = 0.021	*p* = 0.033, $\eta_{p}^{2}$ = 0.059
N-back: accuracy rate	*p* < 0.001, $\eta_{p}^{2}$ = 0.261	*p* = 0.634, $\eta_{p}^{2}$ = 0.008	*p* = 0.403, $\eta_{p}^{2}$ = 0.016

	Simple effect: the control group	Simple effect: the translation group	Simple effect: the interpreting group

Color-shape: switch cost	*p* = 0.566, *r* = 0.086	*p* = 0.806, *r* = 0.049	*p* < 0.001, *r* = 0.492
N-back: global RTs	*p* = 0.546, *r* = 0.092	*p* = 0.051, *r* = 0.332	*p* = 0.002, *r* = 0.455

As can be seen in **Table 6**, the main effect of Test Time was significant for the index of global RTs in all three tasks, and also for the index of accuracy rate in the N-back task, reflecting a general training or test practice effect. For the other indices (Stroop effect, inhibition, facilitation; color-shape mixing and switch costs), the main effect of Test Time was only significant for switch cost. No main effect of Participant Group was found. However, the interaction effect was significant for the two indices of switch cost and N-back global RTs, which requires further simple effect analyses. The lower part of **Table 6** displays *the results of Test-Time simple effect analyses*, which shows that the interpreting group made significant progress in these two indices (*p* < 0.001, *r* = 0.492; *p* = 0.002, *r* = 0.455), and that the translation group made marginally significant progress in N-back RTs (*p* = 0.051, *r* = 0.332) and no significant progress in switch cost (*p* = 0.806, *r* = 0.049), while the control group didn’t make any significant progress (*p* = 0.566, *r* = 0.086; *p* = 0.546, *r* = 0.092). *These results are consistent with the hypothesis that interpreting enhances switching and updating abilities, and that compared with general L2 training, interpreting brings advantages in switching and updating.*

Since all the indices were comparable in the pre-test, we conducted further analysis with the post-test data from the two critical indices of switch cost and N-back RT (as in a cross-sectional design). As **Table 5** shows, significant group differences were found in post-test switch cost and N-back global RTs (*p* = 0.049, *p* = 0.032). The Tukey HSD *post hoc* tests in switch cost showed significantly less switch cost from the interpreting group than the control group (*p* = 0.042, *r* = 0.266), while no significant group difference was found between the control and translation groups (*p* = 0.678, *r* = 0.091), or between the translation and interpreting groups (*p* = 0.280, *r* = 0.189). The Tukey HSD *post hoc* tests in N-back RTs also showed a significant difference between the interpreting and control groups (*p* = 0.040, *r* = 0.253), while only marginal difference was found between the control and translation (*p* = 0.085, *r* = 0.243) and no difference between the translation and interpreting groups (*p* = 0.981, *r* = 0.023). These results further reflect *an interpreting experience advantage in switching and WM updating (compared with general L2 training and translation training).*

## Discussion

The present longitudinal study was intended to investigate whether the two specific bilingual experiences of written translation and consecutive interpreting would produce different cognitive control effects in young adults. To better control potential confounding factors and to avoid the cause-effect ambiguity, we conducted a longitudinal study with three groups of participants matched at the pre-test. The results indicate that the interpreting experience produced significant cognitive advantages in switching (as shown in switch cost in the color-shape task) and updating (as shown in global RTs in the n-back task), while the translation experience produced marginally significant improvements in updating. Neither interpreting nor translation experience brought any advantage to inhibitory control (as shown in the Stroop effect) and monitoring (as shown in global RTs in the number stroop and color-shape tasks, and in mixing cost in the color-shape task).

The present study seems to have provided an answer to the question of “when” practice in one domain generalizes to another domain (part of questions asked by Hartsuiker, 2015). As summarized above, we found that the language switching practice in interpreting (32 class hours in one semester) produced significant domain-general switching advantage, while the language switching practice in translation did not (although there seemed a small tendency of similar effect in switch cost). Since the two language switching experiences of interpreting and translation mainly differ in time pressure and processing demands, this finding of the present study suggests that *a prerequisite for a certain training to bring about general cognitive advantage is probably high processing demand, which is immediate processing under time pressure in the present study*. This is consistent with the speculation made by Schroeder and Marian (2016). That is, when the supply is below the demand in a certain task, the cognitive system tries to adapt and thus gets strengthened. This may explain what has been found in previous studies on bilingual advantages. In other words, bilingual advantages would probably occur if the bilingual task is demanding enough. If, however, a student learns a second language occasionally or once in a while in a classroom, bilingual advantages would probably not occur. This prerequisite for cognitive advantage transfer as defined above may also explain what has been found in non-linguistic practice. Anguera et al. (2013), an excellent example, found that by playing a (high interference) multitasking video game, older adults (60–85 years old) significantly reduced multitasking costs compared to an active control group playing a single task game and a control group without contact with video games. What’s critical is that this training produced benefits to untrained cognitive control abilities, i.e., enhanced sustained attention and WM. In other words, the cognitive advantage transfer (“reduced multitasking costs” to “enhanced sustained attention and WM) was made possible by the multitasking video game, which is certainly more demanding than the single task game.

The present study helps specify relevant findings in the literature. First, previous studies on the relationship between interpreting experience and cognitive control advantages did not explicitly distinguish between the oral and written modes of language switching experience (e.g., Yudes et al., 2011; Dong and Xie, 2014; Woumans et al., 2015). We may now speculate that it was probably the oral mode of language switching experience, i.e., oral interpreting, that had brought the cognitive advantages, especially the advantage in switching (because oral interpreting requires immediate processing under time pressure and is therefore more demanding). Second, the absence of inhibition and monitoring advantages in the present study are consistent with what has been found in relevant previous studies using similar tasks (Yudes et al., 2011; Dong and Xie, 2014). Woumans et al. (2015), however, was a different study that investigated how interpreters, bilinguals and monolinguals performed in the Simon and ANT tasks. The interpreters outperformed the unbalanced (but not balanced) bilinguals in the two tasks (i.e., higher accuracy in both tasks and smaller error congruency effect in the ANT), suggesting the modulation effect of interpreting experience on non-linguistic inhibition tasks. But a closer look at the data indicates that L2 proficiency may have partly contributed to the interpreters’ better performance, since the gap in L2 proficiency between the unbalanced group and the interpreters was large while that between interpreters and balanced bilinguals was small (L2 proficiency on a 5-point scale is 2.6 for unbalanced bilinguals, 3.7 for interpreters and 4.2 for balanced bilinguals; L2 fluency is 5.9 for unbalanced bilinguals, 14.0 for interpreters and 12.9 for balanced bilinguals). To test whether interpreting experience would lead to better non-linguistic inhibition, we may have to conduct more research with more tasks, especially tasks of higher sensitivity (e.g., the Go/Nogo task with ERP techniques).

A challenge for the present study is that two previous studies (Babcock and Vallesi, 2015; Becker et al., 2016) found that professional SIs exhibited reduced mixing costs in the color-shape task when compared to bilingual controls, suggesting that interpreting experience enhances the function of monitoring rather than that of switching. Apart from the criticisms aimed at cross-sectional studies, there may be other reasons to explain the different findings between the present study and the two previous studies. The most probable reason, according to our understanding, lies in the stages of interpreting experience that are different among the studies. At an early stage of interpreting experience as investigated in the present study, switching efficiently between two languages is probably the most obvious challenge, while at a professional stage as investigated in the two previous studies, switching is probably no longer so challenging. Instead, interpreting as a professional (esp. as a professional SIs) requires better management of the situation, monitoring whatever changes and exchanges in the complex situation of communication, and deciding when and how to step in to help the communication. Facing up to the different main challenges at different stages of interpreting experience may lead to exercises of different cognitive control functions and thus strengthen different functions. This explanation also fits with the fact that the control groups were very different among the studies. The control participants in the present study were intermediate L2 learners, while in these two studies they were highly proficient in both languages and they were probably highly proficient in switching between two languages, esp. for the control group of professional consecutive interpreters and translators in Becker et al. (2016). More empirical research is definitely needed to test the explanation.

The finding about the updating advantage in the present study is an important contribution to the literature (see also Morales et al., 2015). In the N-back task, participants were asked to report whether the currently presented item matched the item presented n items back. It is considered a measure of WM, but empirical research indicates that N-back task performance is only weakly correlated with typical measurements of WM, i.e., the complex span (e.g., reading span) (Redick and Lindsey, 2013). The task of interpreting poses high demands on WM, but how individual differencs in WM affect interpreting performance, and whether interpreting training leads to higher WM are controversial (Dong and Cai, 2015). The present study shows that, compared to general L2 training, interpreting training brought significant improvements to updating in WM, and translation training brought marginally significant improvements to updating in WM. What this finding suggests is that updating is perhaps a better way to measure how WM plays its role in the task of interpreting, and thus a better index for the relationship between WM and interpreting.

In short, the present longitudinal study investigated the influence of translation and interpreting experiences on the development of cognitive control functions. The advantage in the non-linguistic switching tasks yielded by interpreting instead of translation experiences at an early stage of interpreting experience suggests that high-processing demands may be critical to improving cognitive control, which may be able to explain the inconsistent findings in bilingual cognitive control reported so far. This explanation is consistent with what was found in the comparative study of multitasking and single task video games (Anguera et al., 2013), and with the supply demand explanation by Schroeder and Marian (2016). Furthermore, the results from the present study lead us to speculate that there might be a development curve of cognitive control enhancement in multitasking training such as L2 training, interpreting training or video games training. At the beginning, the curve goes up slowly but steadily, but at a certain point where participants have reached a cognitive peak, the curve would start to level off. More importantly, the curve may start to drop off slowly when the training becomes less demanding probably because participants become more proficient and automatic in the task. In other words, a skill that requires lots of controlled processing in the early stages may help enhance cognitive control functions, but when that skill becomes automatic and requires far less controlled processing, the early advantages may dissipate. More empirical studies, i.e., studies of longitudinal nature, studies of training with better controlled designs, studies employing additional experimental methods like ERP or fMRI, are certainly needed to verify these speculations.

## Author Contributions

YD had the idea and design and did most of the writing while YL managed the experiments and analyzed the data and helped with the writing and revision.

## Conflict of Interest Statement

The authors declare that the research was conducted in the absence of any commercial or financial relationships that could be construed as a potential conflict of interest.

## Footnotes

1 Apart from the color-shape task, we decided to use the WCST task in the post-test because relevant studies in the literature (Yudes et al., 2011; Dong and Xie, 2014) used this task in their cross-sectional designs and found an interpreter advantage in the performance of the task. If a post-test group comparison in the WCST in the present longitudinal study was found, it would be a triangulation for the two previous studies. Since the WCST was only tested in the post-test, it was not consistent with the longitudinal design, and it was therefore only reported in Supplementary.

2 In addition, mother education significantly predicted three indices of WCST (global RTs: β = -0.178, *p* = 0.042; overall errors: β = -0.215, *p* = 0.017; perseverative errors: β = -0.207, *p* = 0.022). Self-rated L2 proficiency together with age significantly contributed to WCST previous category errors (self-rated L2 proficiency: β = -0.204, *p* = 0.022; age: β = 0.182, *p* = 0.040).
