# Supplementary material for: Classes in Translating and Interpreting Produce Differential Gains in Switching and Updating
Source: Front Psychol. 2016 Aug 30;7:1297. doi: 10.3389/fpsyg.2016.01297 (PMC5003826; doi:10.3389/fpsyg.2016.01297)
Supplement: Supplementary file 1 [file Data_Sheet_1.DOCX]

**Appendix A: Participants excluded to achieve better pretest group matching**

Table S1 lists the relevant information of each participant that was excluded to achieve better group matching in the pretest. Apart from deleting the highest or lowest values in parent’s education or in age to achieve efficient matching of the participant groups, N-back accuracy rate and Stroop effect were also taken into consideration because there was a tendency for the groups to differ in these two factors (see Table 4). For parents’ education (upper part in the table), this principle of deletion concerns three participants (last participant in each of the three groups in the table) who were excluded to balance N-back accuracy rate and Stroop effect among the three groups at the same time. For age (lower part in the table), the principle also concerns three participants. After these participants were deleted, all the variables were better matched in the pretest (see Table 5).

| Table S1. *Description of participants deleted to have a closer match on parents’ education or on age in the pretest, together with their performance in N-back accuracy rate and Stroop effect* | | | | |
| --- | --- | --- | --- | --- |
|  | Father education | Mother education | N-back  accuracy rate | Stroop effect |
| Control A | lowest | lowest | high | high |
| Control B | lowest | low | high |  |
| Control C | low | lowest |  | high |
| Control D | average | lowest | high | high |
| Control E | low | low | high | high |
| Translation A | highest | highest | high | high |
| Translation B | highest | highest | high |  |
| Translation C | highest | highest |  | high |
| Translation D | highest | average | highest |  |
| Translation E | high | average | highest | high |
| Interpreting A | low | lowest | low | low |
| Interpreting B | low | lowest |  | low |
| Interpreting C | lowest | low | low |  |
| Interpreting D | low | low | low | low |
|  | | | | |
|  | Age | | N-back  accuracy rate | Stroop effect |
| Control A | high | | high | high |
| Interpreting A | lowest | | low | low |
| Interpreting B | low | | lowest | low |
| Interpreting C | low | | low | low |

**Appendix B: Brief report of the WCST in the posttest**

The WCST task was used in the posttest in the present study because it was used in relevant previous studies (Yudes et al., 2011; Dong and Xie, 2014) that found an interpreter advantage in the performance of the task. If a posttest group comparison in the WCST in the present longitudinal study revealed similar advantages, it would be a replicate for the two previous studies, and a triangulation for the switching advantage found in the color-shape task.

***Procedure***. The WCST, used to tap participants’ switching ability, was more or less the same as that used by [Dong and Xie (2014](#_ENREF_21)). In each trial, participants were presented a response card and were asked to match the response card with one of the 4 stimulus cards according to one of the three dimensions of geometric figures that were on the card. One dimension was shape (triangle, star, cross, or circle), one was color (red, green, yellow, or blue), and one was number (one, two, three, or four). The response card changed in each trial, while the four stimulus cards didn’t: A) one red triangle, B) two green stars, C) three yellow crosses, and D) four blue circles. For a possible response card of one green cross, for example, the correct response would be A, B or C respectively if participants followed the criterion of number, color or shape. Participants received feedback on whether their response to each trial was correct or not. The matching/sorting rule would change after a few trials (from 5 to 9), and participants were not informed about the change of the underlying rule. The task was composed of two blocks, with the practice block consisting of 12 practice trials and the experimental block 128 trials. A “+” fixation for 1000 ms was presented before the appearance of the stimulus, which would last for a maximum time of 3000 ms or until participants responded by pressing designated keys (one key for each dimension). After that, the feedback of “correct” or “incorrect” lasting 1000 ms would follow.

***Data trimming***. In the WCST task, the same procedure as that for the color-shape task was followed (except that no wrong responses were excluded), and less than 5% of data was discarded (the control group: post-test, 1.65%; the translation group: post-test, 1.71%; the interpreting group: post-test, 2.07%).

***Results.*** Table S2 displays the posttest data for the WCST performance. There are altogether five indices. The 1st index is global RTs, reflecting monitoring ability in mental set shifting. The 2nd index is the number of completed categories, meaning how many correct categories participants have completed, which ranged from 0 to 9 in the present study. “0” means participants failed to complete at least 5 consecutive correct responses to trials belonging to any category. “9” means that participants had successfully completed at least 5 consecutive correct responses to trials for all the categories. The third index is the number of overall errors. More completed categories and fewer overall errors reflect participants’ higher ability in mental set shifting. Two sub-types of error are also used as indices for mental set shifting ability. One is the perseverative error, meaning participants’ failure to change rules after receiving a negative response. The other is a subcategory of perseverative errors: the previous category error, referring to the error made by adopting the immediately preceding category/rule, reflecting one’s inability to change mental sets. Higher shifting ability is indicated by more completed categories and fewer errors.

| Table S2. *Group means (with SD) and comparisons (p value) of participants’ task performances together with multiple comparisons (p value) in the post-test WCST* | | | | |
| --- | --- | --- | --- | --- |
|  | Control  (n= 37) | Translation  (n= 35) | Interpreting  (n= 44) | *p* value |
| WCST: global RTs | 1411.51 (400.01) | 1390.94 (482.05) | 1369.04 (630.15) | .935 |
| WCST: completed categories | 6.72 (3.49) | 6.37 (2.55) | 8.34 (2.87) | .009 |
| WCST: overall errors | 52.13 (15.06) | 52.82 (14.44) | 46.93 (12.92) | .123 |
| WCST: perseverative errors | 29.78 (14.69) | 29.85 (14.37) | 25.06 (13.18) | .214 |
| WCST: previous category errors | 12.81 (9.32) | 15.31 (11.55) | 11.34 (8.81) | .208 |
|  | Tukey HSD post-hoc multiple comparisons | | | |
|  | control- translation | translation -  interpreting | control -  interpreting | |
| WCST: completed categories | *p=*.868 | *p=*.012 | *p=*.046 | |

One-way ANOVA analysis revealed no group difference in global RTs, *F* (2, 113) =.067, *p=*.935, *η*^2^=.001. However, there was a significant group effect in completed categories, *F* (2, 113) =4.963, *p=*.009, *η*^2^=.081. Tukey HSD post-hoc tests showed that the interpreting group completed more categories than both the control (*p=*.046, r=.248) and the translation group (*p=*.012, r=.340), while no difference was found between the latter two (*p=*.868, r=.059).

***Discussion***. The results *show that the interpreting experience produced better switching ability than the translation or general bilingual experience,* which is further supported by the fewest errors made by the interpreting group (although the difference was not significant). This finding is a replicate of what has been found in Yudes et al. (2011) and Dong and Xie (2014), and a triangulation of the switching advantage found in the color-shape task (although there are differences in the two tasks). What is different here is that there were more indices indicating the effect of interpreting experience in the two previous studies. The reason is probably that participants in these studies had received more interpreting training than those in the present study.
